# A common limiter circuit for opioid choice and relapse identified in a rodent addiction model

**DOI:** 10.1038/s41467-021-25080-x

**Published:** 2021-08-09

**Authors:** Jasper A. Heinsbroek, Giuseppe Giannotti, Mitchel R. Mandel, Megan Josey, Gary Aston-Jones, Morgan H. James, Jamie Peters

**Affiliations:** 1 Department of Anesthesiology, University of Colorado Anschutz Medical Campus, Aurora, CO USA; 2 Department of Pharmacology, University of Colorado Anschutz Medical Campus, Aurora, CO USA; 3 Brain Health Institute, Rutgers University and Rutgers Biomedical and Health Sciences, Piscataway, NJ USA; 4 Department of Psychiatry, Robert Wood Johnson Medical School, Rutgers University, Piscataway, NJ USA

**Keywords:** Decision, Addiction

## Abstract

Activity in numerous brain regions drives heroin seeking, but no circuits that limit heroin seeking have been identified. Furthermore, the neural circuits controlling opioid choice are unknown. In this study, we examined the role of the infralimbic cortex (IL) to nucleus accumbens shell (NAshell) pathway during heroin choice and relapse. This model yielded subpopulations of heroin versus food preferring rats during choice, and choice was unrelated to subsequent relapse rates to heroin versus food cues, suggesting that choice and relapse are distinct behavioral constructs. Supporting this, inactivation of the IL with muscimol produced differential effects on opioid choice versus relapse. A pathway-specific chemogenetic approach revealed, however, that the IL-NAshell pathway acts as a common limiter of opioid choice and relapse. Furthermore, dendritic spines in IL-NAshell neurons encode distinct aspects of heroin versus food reinforcement. Thus, opioid choice and relapse share a common addiction-limiting circuit in the IL-NAshell pathway.

## Abstract (web summary)

The neural circuits underlying rewarding effects of drugs of abuse and natural rewards are not fully understood. Here the authors investigate the role of the infralimbic cortex to nucleus accumbens shell pathway during heroin or food choice in rats.

## Introduction

Opioid addiction is a major public health threat^1^ and understanding the neural basis of opioid choice and relapse is essential to the development of novel treatment interventions. From rodent preclinical models, some components of the opioid relapse circuitry have been established, including projections from the prefrontal cortex to the nucleus accumbens, a proposed final common pathway to relapse. This projection is functionally segregated into a dorsal and a ventral circuit^2,3^. Whereas the dorsal component from the prelimbic cortex to nucleus accumbens core (NAcore) drives relapse for multiple drugs of abuse across multiple models^4^, the function of the ventral projection from infralimbic (IL) cortex to nucleus accumbens shell (NAshell) is less clear. While pharmacological manipulations of the IL and the NAshell suggest a driver function for these individual brain regions in heroin relapse^5–7^, a pathway-specific approach for this circuit has not been explicitly utilized in animal models of opioid seeking. Notably, such an approach has revealed a limiter function for the IL→NAshell pathway for both cocaine and alcohol seeking^8–12^.

By comparison to our knowledge of the neural circuitry of relapse, little is known about the neural circuitry underlying the choice between drug versus natural reward. This is striking given that clinical studies have shown nondrug rewards can prolong abstinence from drug use in humans^13^. Preclinical studies support this in that rodents typically prefer natural rewards over drug rewards under conditions of mutually exclusive choice^14**–**19^. This behavior has been called voluntary abstinence because animals completely refrain from using drugs in the presence of a natural reward option, supporting the use of natural reinforcers in contingency management therapy^20^. While the neural circuitry controlling relapse after voluntary abstinence is beginning to be mapped^21,22^, we know virtually nothing about the neural basis of opioid choice. Importantly, opioid use disorder in humans is characterized by a substantial population of drug users that become dependent on the drug^23,24^ and choose opioids over natural rewards. Thus, we wanted to employ a model wherein individual differences in opioid choice would yield a population average of ~50% choice between drug versus natural reward, allowing us to shift choice behavior with functional brain-site manipulations (e.g., avoiding a floor/ceiling effect in choice behavior).

Here we investigated the role of the IL→NAshell pathway in both opioid choice and relapse in a rat self-administration model that identifies a substantial population of rats that prefer/choose heroin over food reward and results in a population average of ~50% choice for heroin over food. In order to understand whether heroin or food demand predicts choice or relapse behavior, we applied principles from the field of behavioral economics. Relationships between Q_0_ (theoretical consumption when the price is free), alpha (α, demand elasticity), Pmax (maximum price paid before consumption declines), EV (essential value of each reward), and choice and relapse behavior indicated that choice and relapse are conceptually distinct constructs. Consistent with this, pharmacological inactivation of the IL cortex increased heroin choice, but decreased heroin relapse, while having no effect on food relapse. However, targeting the IL cortex outputs to the NAshell specifically using a pathway-specific chemogenetics strategy revealed that this pathway is necessary for limiting heroin choice and heroin relapse, but not food relapse. Furthermore, measures of dendritic spine morphology and density in IL→NAshell neurons differentially correlate with specific variables of heroin versus food motivation. The data support a common limiter function for the IL→NAshell pathway in heroin seeking under both choice and relapse conditions.

## Results

### Heroin is a stronger reinforcer than food

**Fig. 1:**
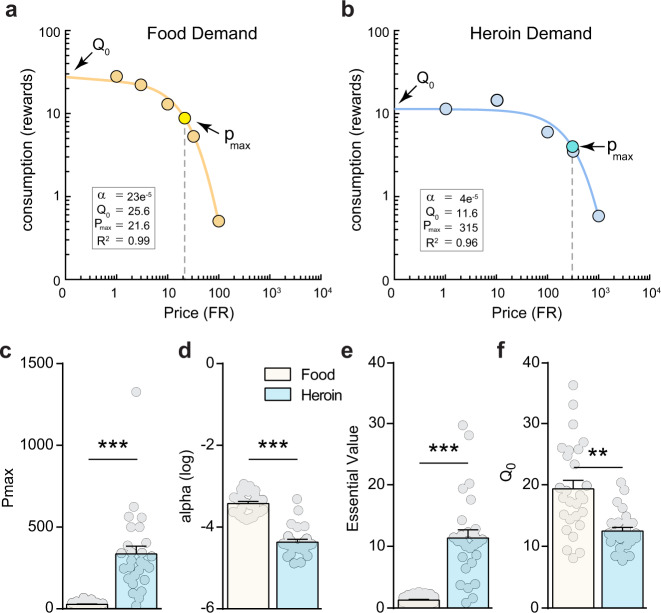
Rats value heroin more than food. Representative demand curves for food (**a**) and heroin (**b**) for a single rat. **c** Rats are more motivated for heroin than for food as assessed by a higher Pmax, the maximum price animals are willing to pay to maintain consumption before levels drop off (two-tailed paired *t*-test, *t*_(26)_ = 6.34, *p* = 5.12 × 10^−7^). **d** Heroin demand is less elastic than food demand (responding drops more rapidly for food as price increases; two-tailed paired *t*-test, *t*_(26)_ = 11.2, *p* = 9.70 × 10^−12^) and correspondingly **e** heroin has a higher essential value than food (two-tailed paired *t*-test, *t*_(26)_ = 7.54, *p* = 2.61 × 10^−8^). **f** Rats consume more food than heroin at low price points as indicated by a higher Q_0_ value (two-tailed paired *t*-test, *t*_(26)_ = 4.87, *p* = 2.40 × 10^−5^). *n* = 27 rats. ***p < 0.001, **p < 0.01 comparing between heroin and food behavioral economic variables. Data were presented as mean ± SEM (**c**–**f**).

Representative demand curves for heroin and food self-administration over increasing price points from a single animal are shown in Fig. 1a, b. Comparing between food and heroin demand for each rat revealed that motivation for heroin is significantly higher as indicated by higher Pmax (Fig. 1c; paired *t*-test: *t*_(26)_ = 6.34, *p* = 5.12 × 10^−7^) and normalized nPmax (Supplementary Fig. 1c; paired *t*-test: *t*_(26)_ = 4.60, *p* = 4.78 × 10^−5^) and Omax values (Supplementary Fig. 1d; paired *t*-test: *t*_(26)_ = 7.64, *p* = 2.10 × 10^−8^). In addition, demand was less elastic for heroin than food as indicated by lower α values (Fig. 1d; paired *t*-test: *t*_(26)_ = 11.2, *p* = 9.70 × 10^−12^). Moreover, the unit-independent EV which allows for direct comparisons between different reinforcers^25^ was significantly higher for heroin (Fig. 1e; paired *t*-test: *t*_(26)_ = 7.54, *p* = 2.61 × 10^−8^). Finally, consumption at low prices (Q_0_) for both heroin and food indicated that rats respond significantly more for food than for heroin under these conditions (Fig. 1f; paired *t*-test: *t*_(26)_ = 4.87, *p* = 2.40 × 10^−5^). Altogether, these results indicate that rats have a higher motivation for heroin and that heroin is considered more essential at higher price points than food reward in this model.

### Heroin choice and relapse are distinct behavioral constructs

**Fig. 2:**
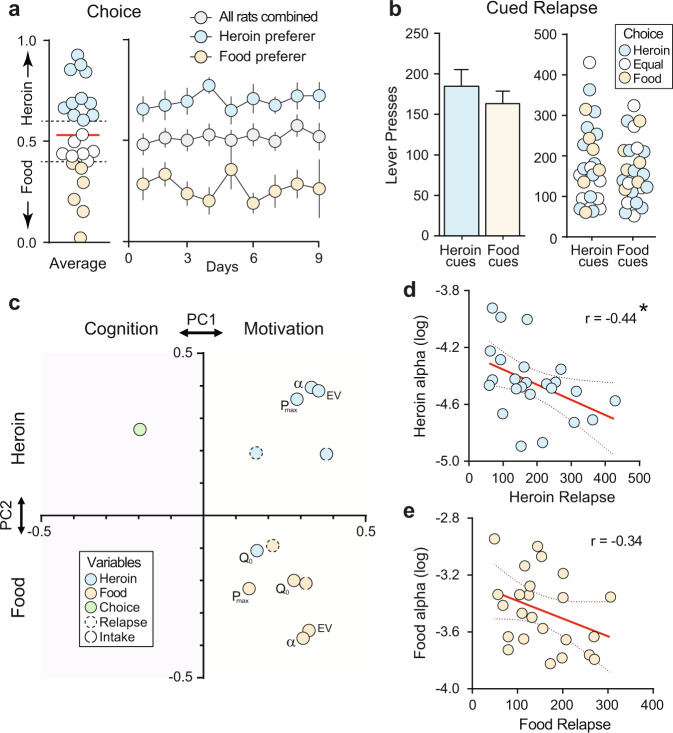
Heroin choice and relapse are distinct behaviors. **a** Overall rats choose food and heroin equally, with a subset of rats preferring heroin (>60% heroin choice, *n* = 11 rats) versus food (>60% food choice, *n* = 6 rats). **b** Rats reinstate food and heroin cues equally, and relapse does not differ between food and heroin preferring animals. **c** Heroin relapse clusters with heroin variables and food relapse with food variables (and heroin Q_0_), but the choice is distinct from these variables in principal component space. Blue colors indicate heroin variables, orange food variables, and choice is labeled green. **d** Heroin α negatively correlates with heroin relapse (Pearson’s *r* = −0.44, *p* = 0.03). **e** Food α does not correlate with food relapse *n* = 24 rats (**a**–**e**). *p < 0.05 significant correlation. Data were presented as mean ± SEM (**a**, **b**).

Following the behavioral economic phase, choice procedures commenced, and rats had to make mutually exclusive choices between heroin versus food. After choice stabilized, a range of choice behavior emerged, with a population average of ~50% heroin choice. This model is ideal to examine the neural basis of choice between heroin and natural reward. To examine the relationship between choice and other behavioral measures, rats were arbitrarily subdivided into Heroin preferers (>60% heroin choice) versus Food preferers (<40% heroin choice) groups (Fig. 2a). Following choice testing and reacquisition of food and heroin self-administration, rats underwent cued relapse tests with each lever presented independently, as during training. No differences were observed in response rates for food versus heroin cues, and relapse did not correlate with heroin choice (Fig. 2b).

We applied a principal component analysis to assess the relationships between behavioral economic variables, total intake of food or heroin, heroin choice, and relapse. Behavioral economic variables, total intake, and relapse clustered along with the first component and separated by reinforcer type (food versus heroin) along with the second component (with a notable exception for heroin Q_0_, which clustered with food variables). By contrast, heroin choice separated along with the first component from all other variables, suggesting that heroin choice is distinct from demand, intake, and relapse (Fig. 2c). We propose that choice may represent a cognitive measure of addiction, whereas the other variables may reflect motivational components. Consistent with prior studies^26**–**28^, heroin relapse (Fig. 2d), but not food relapse (Fig. 2e), negatively correlated with the reinforcer (log) α value, indicating that heroin relapse rates are higher in rats with more inelastic demand. Total intake during self-administration also correlated with behavioral economic variables by reinforcer type (see Supplementary Fig. 2 for a complete correlation matrix), suggesting that motivation for each reinforcer may increase as consumption history increases^29^, or alternatively, that higher motivational drive leads to increased consumption. Corroborating the correlational analysis, statistical comparisons between food preferers and heroin preferers did not reveal any differences between these groups in behavioral economics variables or relapse (Supplementary Fig. 3), with the exception that food preferring rats had elevated Q_0_ compared to heroin preferers (Supplementary Fig. 3e).

### Silencing IL neurons differentially alters heroin choice and relapse

**Fig. 3:**
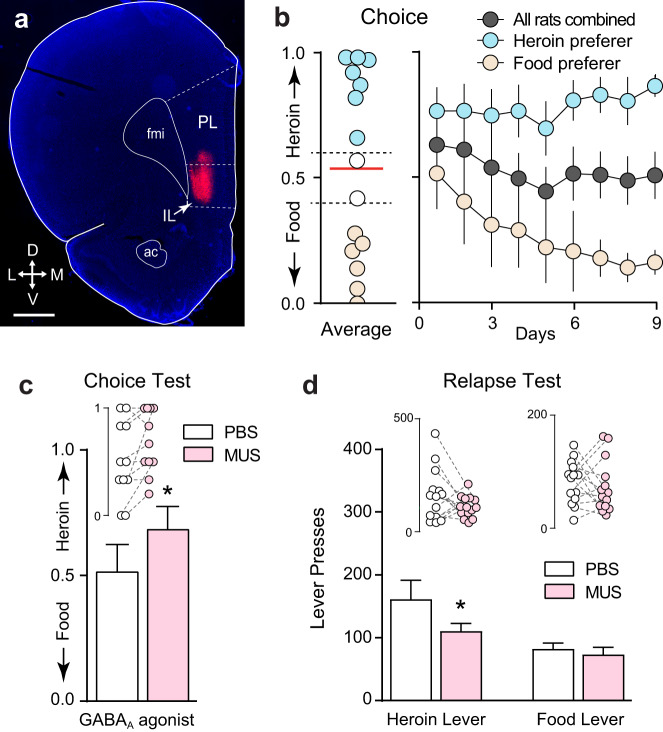
Silencing IL neurons differentially alters heroin choice and relapse. **a** Representative micrograph showing cannula placement and infusion of the fluorescent GABA_A_ receptor agonist muscimol (MUS) into IL (scale bar = 1000 µm). PL prelimbic cortex, fmi forceps minor of the corpus callosum, ac anterior commissure, arrows indicate anatomical orientation along with dorsoventral (DV) and mediolateral (ML) axes. **b** Overall rats choose heroin and food equally, but distinct populations of rats become heroin preferring (*n* = 7) or food preferring (*n* = 6) over time. **c** Muscimol microinjection into IL potentiates heroin preference during choice (two-tailed paired *t*-test, *t*_(10)_ = 2.39, *p* = 0.04; *n* = 11 rats). **d** Muscimol microinjection reduced cued relapse during a test in which both food and heroin levers and cues were present but rewards were unavailable (two-way ANOVA *F*_(1,13)_ = 11.70, *p* = 4.56 × 10^−3^, Bonferroni post hoc test, *p* = 0.04; *n* = 14 rats). *p < 0.05 comparing between vehicle and MUS. Data represented as mean ± SEM.

A separate group of rats was implanted with chronic indwelling cannulae aimed at IL and infused with the GABA-A receptor agonist muscimol (IL-MUS, Fig. 3a) or vehicle (IL-PBS) prior to the choice or relapse tests. This group did not have the extensive training history that the behavioral economics group had, yet opioid choice again stabilized at ~50% (Fig. 3b). Due to the relatively short-lasting effects of intracranial MUS^30^, analyses were restricted to the first hour of the (3 h) choice sessions on test days. IL-MUS significantly shifted choice toward heroin (Fig. 3c; paired *t*-test: *t*_(10)_ = 2.39, *p* = 0.038). Following choice testing and reacquisition of food and heroin self-administration, rats underwent cued relapse tests with both levers present simultaneously, as during training. Relapse rates for heroin cues were significantly higher than those for food cues (Fig. 3d; two-way RM-ANOVA main effect of lever: *F*_(1,13)_ = 11.70, *p* = 0.005). However, once again, there were no differences in relapse rates between heroin versus food preferers (Supplementary Fig. 4), reinforcing the notion that choice and relapse are distinct behavioral constructs. Interestingly, IL-MUS reduced heroin relapse (Fig. 3d; Bonferroni post hoc *p* = 0.038), but not food relapse. Thus, endogenous activity in IL cortex limits opioid choice but drives relapse.

### The IL→NAshell pathway limits both heroin choice and relapse

**Fig. 4:**
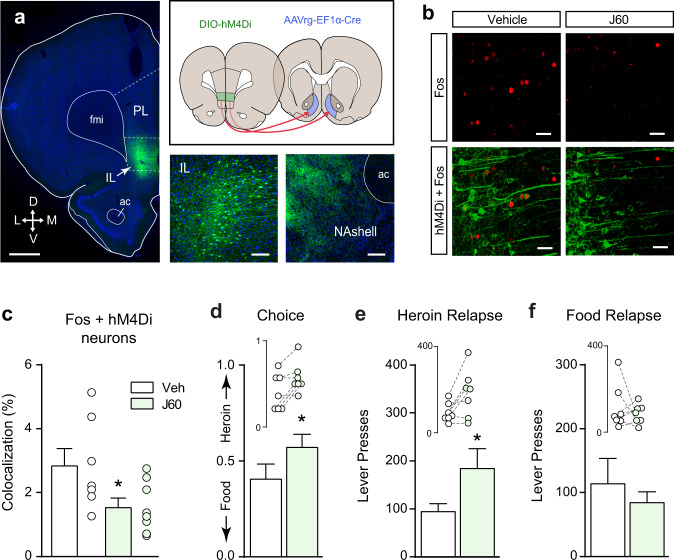
Inhibiting neural activity in the IL→NAshell pathway increases heroin choice and relapse. **a** Representative micrograph showing Gi-DREADD (hM4Di) expression in the IL→NAshell pathway and the spread of virus injections in both IL and NAshell (scale bar = 1000 µm). Only rats with virus expression confined to IL were included in the study (*n* = 7). Inserts show high magnification (20x) DREADD expression in the IL (left; scale bar = 100 µm) and (10x) labeling of IL axons in the NAshell (right; scale bar = 200 µm). PL prelimbic cortex, fmi forceps minor of the corpus callosum, ac anterior commissure, arrows indicate anatomical orientation along with dorsoventral (DV) and mediolateral (ML) axes. **b**, **c** Chemogenetic inhibition of IL→NAshell neurons with the DREADD agonist J60 reduces cocaine-induced Fos expression in virus expressing neurons (two-tailed unpaired *t*-test, *t*_(13)_ = 2.23, *p* = 0.04; vehicle: *n* = 7, J60: *n* = 8 rats; scale bar (**b**) = 30 µm). **d** Inhibition of the IL→NAshell pathway shifts choice towards heroin (two-tailed paired *t*-test, *t*_(6)_ = 3.19, *p* = 0.02). **e** Inhibition of the IL→NAshell pathway does not affect food relapse. **f** Inhibition of the IL→NAshell pathway potentiates heroin relapse (two-tailed paired *t*-test, *t*_(6)_ = 2.73, *p* = 0.03. *n* = 7 rats (**d**–**f**). *p < 0.05 comparing vehicle and J60. Data were represented as mean ± SEM.

We used a combinatorial virus approach to restrict the expression of designer receptors activated exclusively by designer drugs (DREADDs) to the IL→NAshell pathway^31,32^. The efficacy of the DREADD ligand J60 to reduce neuronal activity in animals expressing the inhibitory Gi-DREADD (Fig. 4a) was verified in an independent cohort of rats. J60 significantly reduced IL Fos expression induced by a priming injection of cocaine in DREADD-infected neurons (Fig. 4b, c; unpaired *t*-test: *t*_(13)_ = 2.23, *p* = 0.044). Chemogenetic inhibition of the IL→NAshell pathway shifted choice toward heroin (Fig. 4d; paired *t*-test: *t*_(6)_ = 3.19, *p* = 0.018) and increased heroin relapse (Fig. 4e; paired *t*-test: *t*_(6)_ = 2.73, *p* = 0.034), without altering food relapse (Fig. 4f).

**Fig. 5:**
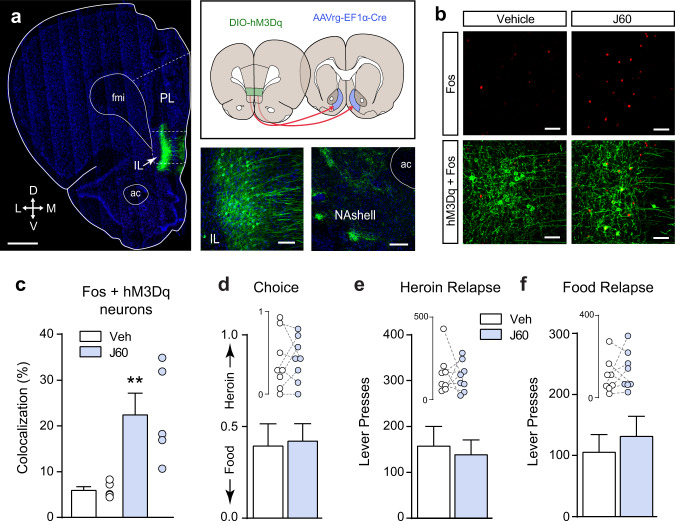
Stimulating neuronal activity in the IL→NAshell pathway does not alter heroin choice or relapse. **a** Micrograph showing Gq-DREADD (hM3Dq) expression in IL→NAshell neurons (scale bar = 1000 µm). Inserts show high magnification (20x) DREADD expression in the IL (left; scale bar = 100 µm) and (10x) labeling of IL axons in the NAshell (right; scale bar = 200 µm). Only rats with virus expression confined to IL were included in the study (*n* = 8). PL prelimbic cortex, fmi forceps minor of the corpus callosum, ac anterior commissure, arrows indicate anatomical orientation along with dorsoventral (DV) and mediolateral (ML) axes. **b**, **c** Chemogenetic stimulation of IL→NAshell neurons with the DREADD agonist J60 induces Fos expression in virus expressing neurons (two-tailed unpaired *t*-test, *t*_(8)_ = 3.51, *p* = 0.01; vehicle: *n* = 5, J60: *n* = 5 rats; scale bar (**b**) = 30 µm). **d** Stimulation of the IL→NAshell pathway does not affect choice or relapse to food (**e**) or heroin (**f**) cues. *n* = 8 rats (**d**–**f**) **p < 0.01 comparing vehicle and J60. Data were represented as mean ± SEM.

The efficacy of J60 to induce neuronal activity in animals expressing the excitatory Gq-DREADD (Fig. 5a) was verified in an independent cohort of rats. J60 significantly induced IL Fos-expression in DREADD-infected neurons (Fig. 5b, c; unpaired *t*-test: *t*_(8)_ = 3.51, *p* = 0.008). However, chemogenetic activation of the IL→NAshell pathway using the excitatory Gq-DREADD did not alter heroin choice (Fig. 5d), nor did it impact heroin relapse (Fig. 5e) or food relapse (Fig. 5f). These data indicate that activity in the IL→NAshell pathway is necessary (Fig. 4), but not sufficient (Fig. 5), to limit heroin choice and heroin relapse. When we employed a standard self-administration, extinction, and cued reinstatement model^9^, chemogenetic activation of the IL→NAshell pathway was sufficient to reduce heroin relapse (Supplementary Fig. 5). Thus, under some conditions, it may be possible to exploit the limiter function of the IL→NAshell pathway to reduce relapse.

### IL→NAshell spines encode heroin choice and relapse

**Fig. 6:**
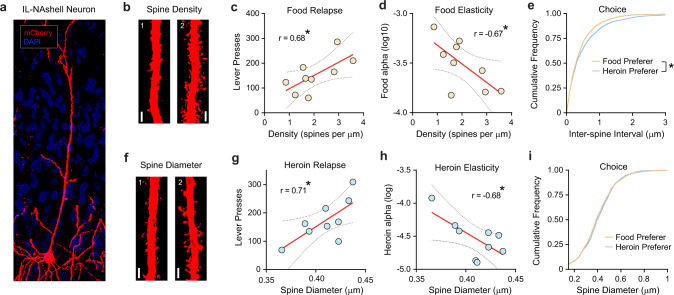
IL→NAshell spines encode heroin choice and relapse. **a** Representative micrograph showing mCherry expression in an IL→NAshell neuron (scale bar = 20 µm). **b** Representative segments with (left) lowest and (right) highest spine density (scale bar = 3 µm). Data were obtained from four dendritic segments in nine rats. **c** IL-NAshell spine density positively correlates with food relapse (Pearson’s *r* = 0.68, *p* = 0.04) and **d** negatively correlates with food alpha (Pearson’s *r* = −0.67, *p* = 0.04). **e** Cumulative frequency distribution showing lower inter-spine interval is associated with food preference (two-tailed two-sample Kolmogorov–Smirnov test, KS2 = 0.07, *p* = 1.29 × 10^−5^). **f** Representative segments showing (left) smallest and (right) largest spine head diameters from IL→NAshell neurons (scale bar = 3 µm). Data were obtained from four dendritic segments in nine rats. **g** Spine head diameter positively correlates with heroin relapse (Pearson’s *r* = 0.71, *p* = 0.03) and **h** negatively correlates with heroin alpha (Pearson’s *r* = −0.68, *p* = 0.04). **i** spine head diameter does not differ between heroin and food preferring rats. Total *n* = 9 rats (food preferer *n* = 4, heroin preferer *n* = 5). *p < 0.05 significant correlation (**c**, **d**, **g**, **h**) or comparing between food and heroin preference (**e**).

At the end of the experiment, structural analyses were performed on dendritic segments from IL→NAshell neurons (using the mCherry tag on the DREADD, Fig. 6a). Spine density (Fig. 6b) and morphology (Fig. 6f) were measured as the number of spines per dendritic segment or the average spine head diameter per dendritic segment, respectively. This analysis revealed bidirectional relationships between these structural measures and heroin versus food reinforcement. IL→NAshell neuron spine density positively correlated with food relapse (Fig. 6c; *r* = 0.68, *p* = 0.043) and negatively correlated with food (log) α value (Fig. 6d; *r* = −0.67, *p* = 0.047). Further analysis of the cumulative frequency distribution of inter-spine intervals (the space between individual spines) revealed a difference between heroin versus food preferers, with food preferers having significantly smaller inter-spine intervals (Fig. 6e; KS2 = 0.07, *p* = 1.29 × 10^−5^). By contrast, spine morphology correlated with measures of heroin, but not food, motivation. Spine head diameter positively correlated with heroin relapse (Fig. 6g; *r* = 0.71, *p* = 0.030) and negatively correlated with heroin (log) α value (Fig. 6h; *r* = −0.68, *p* = 0.043). No differences were observed in the cumulative frequency distribution of spine head diameter between heroin versus food prefers (Fig. 6i), and no other significant correlations were observed besides those reported above.

## Discussion

These results identify the first neural circuit that acts as an endogenous limiter of heroin seeking, namely the IL→NAshell pathway. These results are congruent with findings indicating that this pathway is recruited by extinction learning to limit cocaine seeking^8,9,33^, and suggest that giving animals a choice between drug versus natural rewards, which has been likened to a form of contingency management therapy^18^, may recruit similar circuits as extinction training, another form of cognitive behavioral therapy. Furthermore, these results identify the first neural circuit implicated in opioid choice; specifically the IL→NAshell pathway similarly functions as a limiter of heroin choice (when food is an alternative reward). Correlational and principal components analyses support the notion that heroin choice and relapse are distinct constructs, making it all the more striking that the limiter function of the IL→NAshell pathway extends to both forms of addictive behavior. Nonetheless, other IL outputs may mediate heroin relapse, as global inactivation of the IL cortex did not elicit parallel effects on relapse, as it did on choice.

Behavioral economics has been applied to the study of human substance abuse and was shown, along with choice-based relapse, to relate to measures of dependence and abuse^34^. It has further been suggested that choice (between drug and alternative reward) may reveal human subpopulations that could benefit from contingency management therapy^35**–**37^. Corroborating this, preclinical studies consistently show a negative relationship between α and relapse rates^27,38^, consistent with our findings here for heroin. However, principal component and correlational analyses of behavioral economic variables, choice, and relapse clearly identified choice as a distinct behavioral construct. Whereas behavioral economic variables associated with each other, predominantly clustering by reward type (heroin versus food), they also clustered with relapse and total intake (consumption history). Thus, these variables likely reflect a larger behavioral construct, possibly motivation. By contrast, the opioid choice fell within its own principal components space, which we propose represents the construct of cognition. While purely theoretical, regardless of the hypothetical construct assigned to principal component space in these analyses, heroin choice clearly separated from the other behavioral variables, including heroin relapse.

Since global inactivation of IL cortex with IL-MUS produced a similar upward shift in heroin choice as IL→NAshell pathway-specific inhibition, the opioid choice may predominantly recruit this IL output (NAshell). Similarly, IL→NAshell pathway inhibition produced an increase in heroin, but not food, relapse. Thus, the IL→NAshell functions as a common limiter of these distinct behaviors and may reflect the top-down cognitive control IL exerts over NAshell-motivational processes^39,40^. Though IL→NAshell activation was not sufficient to limit opioid choice or relapse during acute withdrawal after choice procedures, it was capable of reducing cued heroin relapse in a standard extinction-reinstatement model. It is possible that IL→NAshell activity is already at a ceiling after choice procedures and/or acute withdrawal, but may be further enhanced to limit relapse after extinction and/or protracted withdrawal. Interestingly, global IL-MUS inactivation actually decreased heroin, but not food, relapse, an effect opposite that of IL→NAshell pathway-specific inhibition. This suggests that the IL cortex can also function as a driver of heroin seeking, consistent with other reports^5–7^. Nonetheless, our findings in this heroin model are consistent with observations that IL neuronal ensembles exert inhibitory control over alcohol seeking^41,42^. As specific IL outputs were not identified in the latter studies, these apparent discrepancies may relate to different IL outputs being engaged under different conditions.

Surprisingly, very little is known about the neural circuits controlling the choice between opioid versus natural reward. However, choice behavior likely involves distinct prefrontal circuits that attribute and compare the expected value of different rewards, and that monitor behavioral strategies. Within the IL cortex, neuronal ensembles encoding alcohol versus saccharin rewards (two tastant rewards) were found to exhibit some overlap, but an equal portion of these ensembles were distinct^43^. Although the IL cortex has not to our knowledge been examined in the context of opioid choice, within the orbitofrontal cortex, which receives input from the IL cortex^44^, distinct populations of neurons encode food or heroin choice^45^. The adjacent anterior insular cortex and its projection to the central amygdala regulate methamphetamine relapse following voluntary abstinence by food choice^22^, and expression of a ɣ-aminobutyric acid (GABA) GABA transporter (GAT-3) in the central amygdala has been linked to alcohol choice in both rodents and humans^46^. Other anterior insular projections to the NAcore mediate choice between two distinct food rewards, and downstream μ opioid receptors in the NAcore are involved^47^. Mu opioid receptors are also highly expressed in the NAshell, and concentrated in hedonic hot spots which directly mediate opioid and food reward^48**–**50^. Activation of these μ opioid receptors reduces neuronal activity and increases food consumption, an effect that is consistent with other pharmacological means of inhibiting NAshell activity^51,52^. This may in part account for observations that prolonged heroin self-administration, which directly activates μ opioid receptors, increases motivation for both heroin and sweet reward^29^.

Analyses of dendritic spines suggest that specific structural features in IL→NAshell neurons may encode distinct aspects of heroin versus food reinforcement, with spine head morphology correlating with heroin measures (heroin relapse and demand elasticity), and spine density with food measures (food relapse and food elasticity). Opioid choice, by contrast, may specifically relate to the inter-spine interval, with lower opioid choice reflected in more closely spaced spines along the dendritic shaft. How these structural markers relate to the physiological activity of these neurons, and the specificity of these markers to the IL→NAshell pathway remains to be determined. It is nonetheless striking that these post hoc structural analyses distinguished heroin versus food relapse in morphological versus densitometric domains, despite the fact that heroin relapse correlated with food relapse in this study (Supplementary Fig. 2). Notably, however, heroin and food relapse did not correlate in the IL-MUS study (Supplementary Fig. 4). Thus, presenting each lever independently as in the former study may have resulted in greater generalization across levers, even though the order in which they were presented was counterbalanced. This may also explain why heroin and food relapse rates did not differ in the behavioral economics study, but did in the IL-MUS study. When both levers were presented simultaneously as in the latter study, thus forcing the rats to choose how they allocated their seeking behavior, heroin relapse rates were higher than those for food.

In our choice model, we identified subpopulations of heroin preferring versus food preferring rats under equivalent price conditions (FR3). This contrasts with findings from other choice models, which have been dubbed voluntary abstinence models^18,53^ due to the majority of rats exclusively choosing food reward over drug reward. Still, others have been able to produce these subpopulations by increasing the delay to food reward (i.e., discounting food)^54^ or by extending the duration of daily drug self-administration (e.g., using long-access procedures)^55**–**57^. Our model yields ~50% heroin choice (without altering conditions between food and heroin) regardless of whether we used a behavioral economics model where animals had an extensive opioid self-administration history versus an abbreviated choice model with a more limited history of opioid exposure. This affords us a unique opportunity to examine neurobiological differences in these subpopulations going forward. Future studies should examine how the IL→NAshell pathway fits within a broader circuitry controlling choice between opioid and natural reward, and further probe the circumstances under which this pathway is capable of limiting opioid addiction-like behaviors.

## Methods

### Animals

All animal procedures followed guidelines approved by the University of Colorado Denver, Anschutz Medical Campus Institutional Animal Care and Use Committee. Subjects were age-matched (P55-60) male and female Wistar rats (Charles River, Raleigh, NC). Animals were single-housed in a temperature and humidity-controlled environment (lights on 7 a.m.–7 p.m.) with free access to standard laboratory chow and water. Our procedures followed the guidelines outlined in the Guide for the Care and Use of Laboratory Animals^58^. Rats were excluded from the final dataset if catheter patency was lost, if they were identified as outliers during statistical analysis (see Statistical Analyses), if they failed to meet specified behavioral criteria, or for poor placement and/or virus expression (where relevant).

### Surgical procedures

Rats were anesthetized with ketamine/xylazine (80/7 mg/kg) and implanted with an intravenous catheter in the right jugular vein and intracranial cannulas (Plastics One, Roanoke, VA, USA)^9,59^ using the following coordinates relative to bregma: NAshell +1.4 mm anterior-posterior (A/P), ±0.8 mm mediolateral (M/L), and −6.0 mm dorsoventral (D/V); IL +2.9 mm A/P, ±0.6 mm M/L, and −2.5 mm D/V. Carprofen (5 mg/kg) and cefazolin (30 mg/kg) were administered to alleviate surgical pain and prevent infection. Rats were allowed to recover from surgery at least 1 week prior to virus infusions.

### Chemogenetics approach

Under light isoflurane anesthesia, rats received bilateral injections of the virus into IL and NAshell (volume: 0.5 µl/side, rate: 0.1 µl/min). Injectors extended 3 mm beyond cannula for IL and 2 mm for NAshell. The NAshell was injected with a retrogradely transported AAV vector carrying Cre recombinase (AAVrg-Ef1α-Cre; *n* = 30; Salk Institute; titer 1.1 × 10^12^ GC/ml). The IL was injected with viral vectors carrying inhibitory or excitatory designer receptors exclusively activated by designer drugs (Gi- or Gq-DREADDs; AAV2-hSyn-DIO-hM4Di-mCherry; *n* = 15; Addgene #44362-AAV2; titer 3.5 × 10^12^ GC/ml or AAV2-hSyn-DIO-hM3Dq-mCherry; *n* = 15; Addgene #44361-AAV2; titer 3.5 × 10^12^ GC/ml). The DREADD ligand JHU37160 dihydrochloride (J60, HelloBio #HB6261), dissolved in sterile water, was administered at a dose of 0.1 mg/kg (1 ml/kg, IP) 15 min prior to testing^60^.

### Intracranial muscimol microinjections

Rats received bilateral microinjections of muscimol (MUS; volume: 0.3 µl/side, rate: 0.25 µl/min) or vehicle (sterile PBS) into the IL cortex through their intracranial cannula just prior to testing. Injectors extended 3 mm beyond the cannula. The dose of MUS (15 ng/side) was based on previous work indicating this dose does not produce locomotor effects^61^. The order of MUS versus vehicle alternated between within-subjects tests. At the end of the experiment, rats were microinjected with an equimolar, equi-volume fluorescent-tagged MUS (MUS-TMR-X) prior to histological procedures in order to visualize the spread of MUS-TMR-X at the injection site^62^.

### Food self-administration for behavioral economics

Food self-administration training (1 h/session/weekday) occurred in standard operant chambers inside ventilated, sound-attenuating cubicles (Med Associates, St. Albans, VT, USA). Rats were trained to self-administer food pellets (Bio-Serv, #F0165, 45 mg) on a fixed-ratio 1 (FR1) schedule of reinforcement. During food self-administration (1 h/session/weekday), only the right retractable lever was extended. Three pellets and a 3.5 kHz tone cue (5 sec) were delivered each time the FR requirement was reached, and the lever retracted during cue presentation. After five sessions, training progressed through five additional FR schedules (FR3, FR5, FR10, FR32, and FR100) with at least 2 days on each FR, or until ≤20% variability in rewards earned was achieved.

### Heroin self-administration for behavioral economics

After completing food self-administration, rats began daily heroin self-administration training (FR1; 3 h/session/weekday), during which only the left lever was extended. Heroin (diamorphine HCl; NIDA Drug Supply Program) was dissolved in 0.9% sterile saline. After an FR requirement was reached, a heroin infusion (0.04 mg/50 µl/2.85 sec) was delivered, a light above the left lever was turned on (5 sec), and the lever retracted during cue presentation. After at least five sessions, animals progressed over additional FR schedules (FR3, FR10, FR21, FR32, FR45, FR100, FR316, FR1000), with at least 2 days on each FR, or until ≤20% variability in rewards earned was achieved. To prevent infection and catheter occlusion, cefazolin and taurolidine-citrate solution were administered after each self-administration session.

### Generating demand curves for behavioral economics

We used a between-session behavioral economics paradigm where male rats were allowed to self-administer food or heroin in daily sessions of fixed duration^28^. Food and heroin demand curves were generated separately for each animal in Matlab (Mathworks, R2019b), using the average responding over the last two sessions on each FR, according to the formula^63^:

1 $${{{{{\rm{ln}}}}}}Q={{{{{\rm{ln}}}}}}{Q}_{0}+k({e}^{-\propto {Q}_{0}C}-1)$$

C indicates consumption (i.e., number of pellets earned or heroin infusions received at each price-point, see ref. ^38^:) and constant k (set to 8.85) specifies the range of consumption values. From the resulting demand curves, we extracted the following variables: α (demand elasticity; rate at which consumption declines with increasing price), Q_0_ (consumption at null cost; intrinsic motivational efficacy of the reward), Pmax (maximum price paid before demand rapidly declines; derived using a solver algorithm), and Omax (the maximum rate of responding to defend desired consumption)^28,63,64^. We also calculated EV according to the formula^25^:

2 $$\,{{{{{{\mathrm{EV}}}}}}}={(100\cdot \alpha \cdot {k}^{1.5})}^{-1}$$

Because EV is independent of reinforcer magnitude^25^, it is useful for comparing different reinforcers directly. We also compared normalized Pmax values between food and heroin demand by dividing Pmax by Q_0_ values, as described previously^64^.

### Choice procedures after behavioral economics

Choice procedures were implemented at the end of heroin self-administration in the behavioral economics study. During this phase, rats were given 14 trials to choose food (three pellets + food-tone) or heroin (0.04 mg infusion + heroin-light). During each trial, both right and left levers were extended, and the rat needed to reach an FR3 requirement on one of the levers to earn a food or heroin reward. Afterward, both levers retracted for a 10 min time-out between trials (house-light off and levers retracted). The first five-choice sessions contained two forced-choice trials for each reinforcer (four total trials) during which only one lever was extended and ten free-choice trials with two levers. Forced choice trials were administered in the order: food-heroin-food-heroin. Afterward, rats moved to free-choice sessions (both levers extended for all 14 trials) for the remainder of choice training. Following at least three free-choice sessions or until there was ≤20% variability in total choices over the last two sessions, rats started choice tests, separated by at least two stable choice sessions. Prior to each test, rats were injected with J60 or vehicle in a randomized, counterbalanced order. The choice was expressed as the ratio of heroin choices over total choices.

### Abbreviated self-administration choice protocol

For the IL-MUS experiment, we used a protocol wherein male and female rats were trained to self-administer food and heroin simultaneously during daily self-administration sessions. The parameters were equivalent to those described above for the behavioral economics experiment, except that sessions were a total of 3 h long, and the number of self-administration days was held constant across all rats. After six sessions on an FR1 schedule of reinforcement, rats progressed to an FR3 scheduled for three sessions and then proceeded to free-choice sessions thereafter. These free-choice sessions were identical to those used after behavioral economics procedures except the session duration was held constant at 3 h (e.g., not capped at 14 trials). After at least nine sessions on choice, and meeting stability criteria (≤20% variability in total choices over the last two sessions), rats began testing after intra-IL microinfusion of the vehicle or MUS (see Intracranial muscimol microinfusions). Rats underwent two choice sessions in between test days using a within-subject design with treatment order counterbalanced across subjects.

### Cued relapse testing

After rats completed choice testing, they were returned to self-administration procedures to ensure recent exposure to both heroin and food (and their respective cues) across all rats (including heroin and food preferers). After three FR3 reacquisition sessions, rats began cued relapse testing. Thus, the relapse terminology used throughout this manuscript refers specifically to relapse during acute withdrawal (except Supplementary Fig. 5). For the behavioral economics study, each lever was extended separately in two 30-min bins, separated by a 10 min time-out (levers retracted, house-light off). Pressing on each lever resulted in cue delivery (food-tone or heroin-light) on an FR3 schedule. Levers retracted during cue delivery, as during self-administration sessions. The order in which the food or heroin lever was presented was counterbalanced across rats and tests. For the IL-MUS experiment, the relapse test was conducted with both levers (food and heroin) available simultaneously for the 1-h test session. Each rat received drug (J60 or IL-MUS) or vehicle treatments in a randomized, counterbalanced order separated by at least two FR3 reacquisition sessions.

### Histology and immunohistochemistry

Rats were transcardially perfused with 0.9% saline followed by 10% formalin. Brains were extracted, post-fixed in formalin, cryoprotected in 20% sucrose, and sectioned (40 µm) on a cryostat (Leica, CM-1950). Free-floating sections were blocked in 2% normal donkey serum (NDS) in phosphate-buffered saline with 0.3% Triton X-100 (PBS-T) and incubated overnight at 4 °C with rabbit anti-cFos (1:2000, Millipore Cat# ABE457, RRID: AB_2631318) and/or chicken anti-mCherry (1:5000, LifeSpan, Cat# LS-C204825, RRID: AB_2716246). Sections were then rinsed in PBS-T and incubated for 2 h in PBS-T with 2% of NDS containing fluorescent Alexa-conjugated secondary antibodies (1:500, Jackson ImmunoResearch Labs). NeuroTrace fluorescent Nissl 640/660 (1:500, Thermo Fisher Scientific, RRID:AB_2572212) was applied during the secondary antibody step, or DAPI coverslipping media was used as a counterstain. Images were acquired with an Olympus slide scanner microscope (VS120; 2x or 10x air objective; Olympus, Center Valley, PA). Ten rats were excluded from chemogenetics experiments based on low or misplaced virus expression.

### Cell counts

The chemogenetic approach was validated using Fos as a measure of neuronal activity after J60 or vehicle administration in male and female rats. The Gi (hM4Di)- or Gq (hM3Dq)- DREADD was restricted to the IL-NAshell pathway using the combinatorial viral strategy described above (see Chemogenetics approach). To verify Gi-DREADD functionality, rats received an injection of cocaine (cocaine hydrochloride, 10 mg/kg, 1 ml/kg IP, NIDA Drug Supply) to induce Fos, 15 min after J60 (0.1 mg/ kg, 1 ml/kg) or sterile water pretreatment. To verify Gq-DREADD functionality, rats received J60 (0.1 mg/kg, 1 ml/kg IP) or vehicle (sterile water). All injections were given in the home cage, and all rats were perfused transcardially 2 h after J60 or vehicle. Histology and immunohistochemistry were performed as described above, and images were analyzed using Imaris software (Bitplane). A region of interest (ROI) was set and the number of Fos+, hM4Di−, or hM3Dq-mCherry+ neurons in the IL was automatically quantified using the Imaris spot detection function on thresholded images. The number of co-expressing neurons in each section were determined using the colocalize spot function. For all rats, the number of cells was averaged within each hemisphere across at least three sections.

### Dendritic spine analyses

Spine analyses were performed in DREADD-infected, mCherry-tagged neurons from the behavioral economics study^65^. Dendritic segments from IL→NAshell neurons selected for spine analysis were visually connected to the soma. Z-series were acquired with a confocal microscope (Olympus FV1200) using a 60x oil immersion objective (1.4 NA), at 1024 × 512 frame size using 4.1x digital zoom and a 0.1 µm step size. mCherry was excited using a 488 nm argon-ion laser line and overall, imaging parameters were held constant during the acquisition process. Apical dendrite segments from layer 5/6 pyramidal neurons were imaged in layer 2/3 (~300 µm from the soma), ~10 µm before branching into the apical tuft. Images were cropped and deconvolved using Huygens (SVI) software. Dendritic segments (mean ± SEM length: 65.6 ± 0.38 µm) were imported into Imaris (Bitplane) software and spines were semiautomatically drawn using the filament module. Spine density and head diameter values were determined from four dendritic segments per animal.

### Statistical analyses

All statistical tests were performed in Prism (GraphPad, 8.0) and Matlab (Mathworks, 2019b). Choice and relapse tests were analyzed using repeated measures analysis of variance (ANOVA), followed by Bonferroni corrected planned comparisons, or by using two-tailed, planned comparison paired *t*-tests. Cell counts for Fos expression analyses were analyzed using two-tailed unpaired *t*-tests. Linear relationships between variables were analyzed using Pearson’s Correlation Coefficient. Principal component analysis was conducted using z-transform scaled data in Matlab. Cumulative frequency distributions were compared using two-sample Kolmogorov–Smirnov tests in Matlab. Outliers were identified using ROUT analysis. Significance was defined as alpha <0.05 and data were graphed as mean ± standard error of the mean (SEM).

### Reporting Summary

Further information on research design is available in the Nature Research Reporting Summary linked to this article.

## Supplementary information

Supplementary Information

Reporting Summary

## Data Availability

The datasets generated during and/or analysed during the current study are available from the corresponding author on reasonable request. Source data are provided with this paper.

## Source data

Source Data
