# Supplementary material for: A common limiter circuit for opioid choice and relapse identified in a rodent addiction model
Source: Nat Commun. 2021 Aug 9;12:4788. doi: 10.1038/s41467-021-25080-x (PMC8352904; doi:10.1038/s41467-021-25080-x)
Supplement: Supplementary file 1 — Supplementary Information [file 41467_2021_25080_MOESM1_ESM.pdf]

Supplementary Figure 1

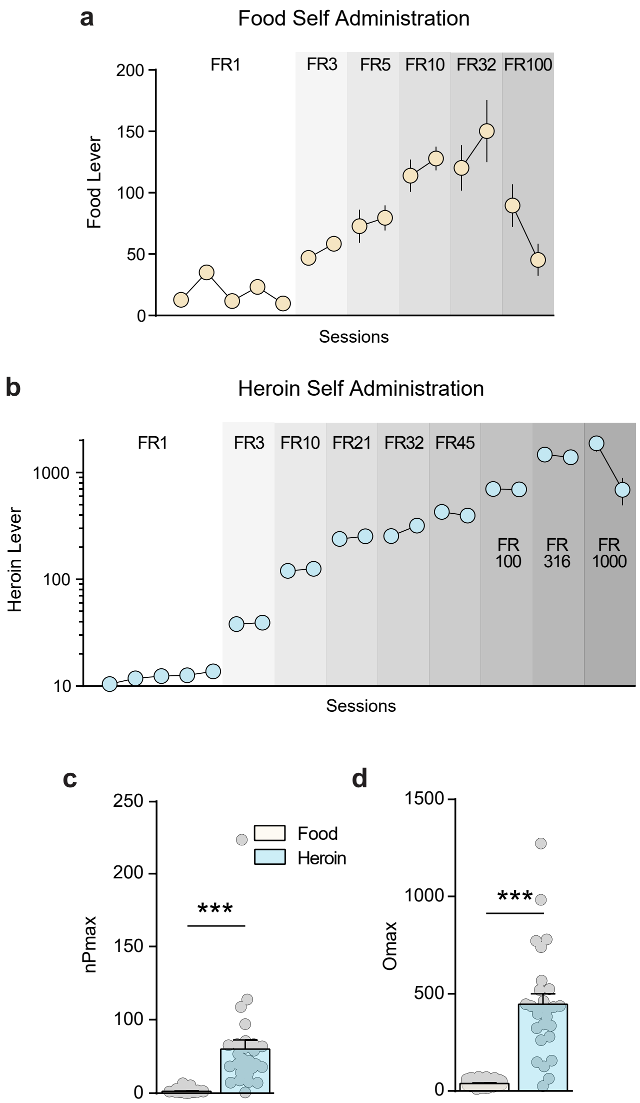

Supplementary Figure 1. Heroin and food self-administration in the behavioral economics study. (a) Acquisition of food self-administration in rats. The first and last day of responding are shown for each schedule for behavior economics analysis. (b) Acquisition of heroin self-administration showing first and last day of responding for each schedule following FR1. (c) Rats are more motivated for heroin than for food as assessed by a higher normalized nPmax values (Pmax divided by Q_0; two-tailed paired t-test; $t_{(26)} = 4.60$, $p = 4.78 \times 10^{-5}$), the maximum price animals are willing to pay to maintain consumption before levels drop off. (d) Rats respond more to defend desired heroin than desired food levels as indicated by Omax, the maximum rate of responding at increasing price points before responding drops off (two-tailed paired t-test; $t_{(26)} = 7.64$, $p = 2.10 \times 10^{-8}$). n = 27 rats. *** $p < 0.001$ comparing between heroin and food behavioral economic variables. Data are presented as mean ± SEM.

Supplementary Figure 2

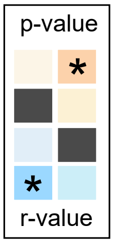

		Heroin							Food					
		Q_0	α	EV	P_{max}	Cue Test	Total Intake	Choice	Q_0	α	EV	P_{max}	Cue Test	Total Intake
Heroin	Q_0		0.61	0.75	0.09	0.80	0.01	0.06	0.10	0.58	0.74	0.84	0.48	0.43
	α	-0.11		$3e^{-11}$	$5e^{-6}$	0.03	$6e^{-5}$	0.99	0.90	0.78	0.98	0.94	0.57	0.78
	EV	0.07	-0.93		$2e^{-8}$	0.09	$8e^{-5}$	0.81	0.39	0.88	0.95	0.70	0.65	0.31
	P_{max}	-0.35	-0.79	0.88		0.28	0.02	0.37	0.82	0.93	0.69	0.97	0.65	0.34
	Cue Test	0.05	-0.44	0.35	0.23		0.30	0.33	0.52	0.86	0.75	0.40	0.02	0.47
	Total Intake	0.51	-0.73	0.72	0.47	0.22		0.12	0.21	0.50	0.48	0.98	0.66	0.23
	Choice	-0.40	$-3e^{-3}$	0.05	0.19	0.21	-0.33		0.06	0.07	0.11	0.32	0.63	0.15
Food	Q_0	0.34	-0.03	0.18	0.05	-0.14	0.27	-0.39		0.06	0.04	0.15	0.66	$6e^{-7}$
	α	-0.12	-0.06	0.03	-0.02	-0.04	-0.15	-0.38	-0.39		$2e^{-14}$	$4e^{-4}$	0.10	0.01
	EV	0.07	0.01	0.01	0.09	0.07	0.15	-0.33	0.43	-0.97		$1e^{-3}$	0.08	$4e^{-3}$
	P_{max}	-0.04	0.02	-0.08	-0.01	0.18	0.01	-0.21	-0.30	-0.66	0.62		0.08	0.71
	Cue Test	0.15	-0.12	0.10	0.10	0.49	0.09	-0.11	0.10	-0.34	0.37	0.36		0.49
	Total Intake	0.17	-0.06	0.22	0.20	-0.15	0.26	-0.30	0.83	-0.50	0.56	-0.08	0.15	

Supplementary Figure 2. Complete correlation matrix comparing all variables in the behavioral economics study. Behavioral economic variables correlate with each other, and with total food or heroin intake for heroin and food demand respectively. In addition, heroin alpha predicts heroin relapse and heroin and food reinstatement are correlated. n = 24 rats. * $p < 0.05$ significant correlations (Pearson's r) compared to the null hypothesis of no relationship between variables.

Supplementary Figure 3

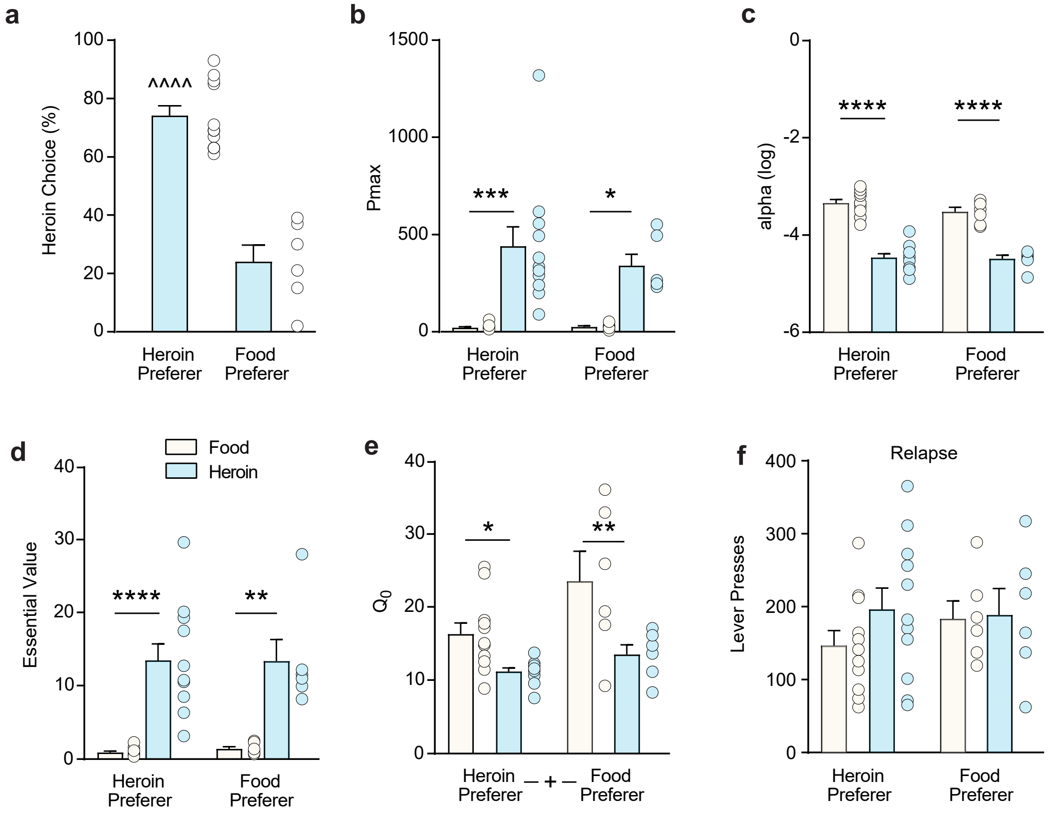

Supplementary Figure 3. Analysis of all variables in the behavioral economics study by heroin versus food preference. (a) Heroin choice is higher in heroin preferers (n = 11) compared to food preferers (n = 6; two-tailed unpaired t-test; $t_{(15)} = 7.89$, $p = 5.07 \times 10^{-7}$). (b) Pmax is higher for heroin than food, irrespective of preference (two-way ANOVA main effect of reinforcer: $F_{(1,15)} = 25.75$, $p = 1.37 \times 10^{-4}$, Bonferroni post-hoc Heroin preferer: $p = 4.02 \times 10^{-4}$, Food preferer: $p = 0.03$). (c) alpha is lower for heroin than food irrespective of preference (two-way ANOVA main effect of group $F_{(1,15)} = 137.7$, $p = 5.86 \times 10^{-9}$, Bonferroni post-hoc Heroin preferer: $p = 4.50 \times 10^{-8}$, Food preferer: $p = 1.31 \times 10^{-5}$). (d) The essential value of heroin is higher than food irrespective of preference (two-way ANOVA main effect of reinforcer: $F_{(1,15)} = 42.76$, $p = 9.37 \times 10^{-6}$, Bonferroni post-hoc Heroin preferer: $p = 9.63 \times 10^{-5}$, Food preferer: $p = 2.45 \times 10^{-3}$). (e) Q_0 is higher for food than heroin irrespective of preference (two-way ANOVA main effect of reinforcer $F_{(1,15)} = 19.10$ $p = 5.49 \times 10^{-4}$, Bonferroni post-hoc Heroin preferer: $p = 0.05$, Food preferer: $p = 5.29 \times 10^{-3}$), and is higher overall in food preferers (two-way ANOVA main effect of group $F_{(1,15)} = 5.26$, $p = 0.04$). (f) Heroin and food relapse rates are similar and do not differ between heroin and food preferers. Heroin preferer n = 11 rats, food preferer n = 6 rats. ^^^^ $p < 0.0001$ comparing between heroin and food preferers. * $p < 0.05$, ** $p < 0.01$, *** $p < 0.001$, **** $p < 0.0001$ comparing between food and heroin reinforcers. + $p < 0.05$ comparing between heroin and food preferers. Data are represented as mean ± S.E.M.

Supplementary Figure 4

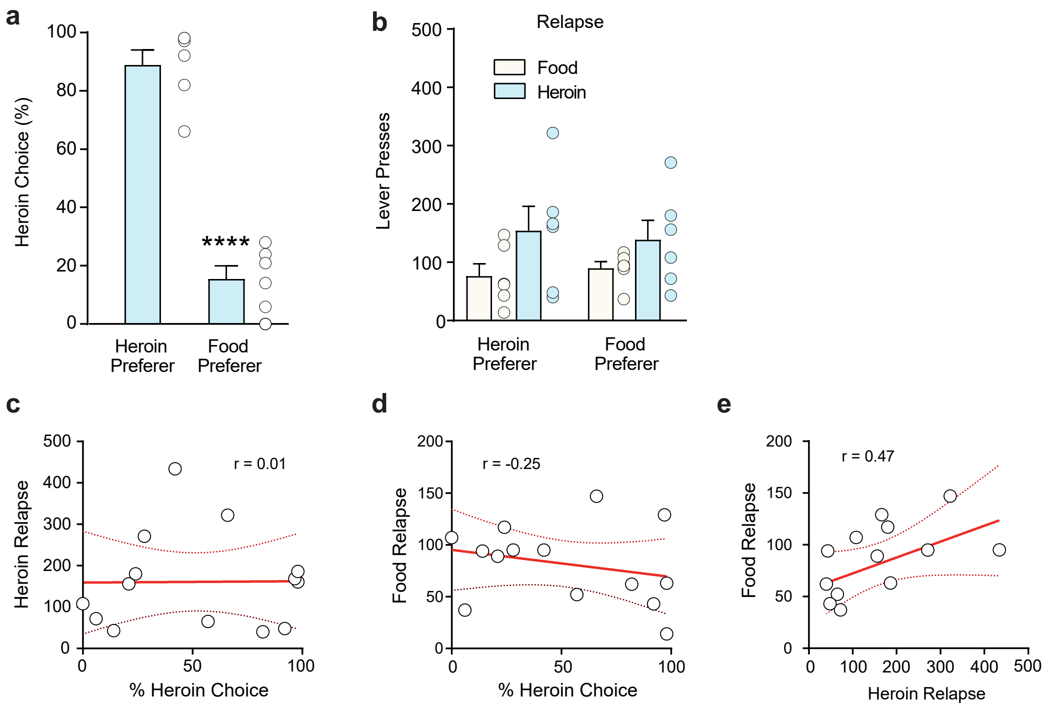

Supplementary Figure 4. Analysis of choice and relapse by heroin versus food preference following an abbreviated self-administration choice protocol. (a) Heroin choice is higher in heroin preferers compared to food preferers (two-tailed unpaired t-test; $t_{(10)} = 10.72$, $p = 4.19 \times 10^{-7}$). (b) Relapse rates do not differ between heroin and food preferers. (c) Choice and heroin relapse or (d) food relapse are not related. (e) Heroin and food relapse are not correlated when cues are presented during the same session. Total n = 14 rats (food preferer n = 6, heroin preferer n = 6). **** p < 0.0001 comparing between heroin and food preferers. Data are represented as mean ± S.E.M.

Supplementary Figure 5

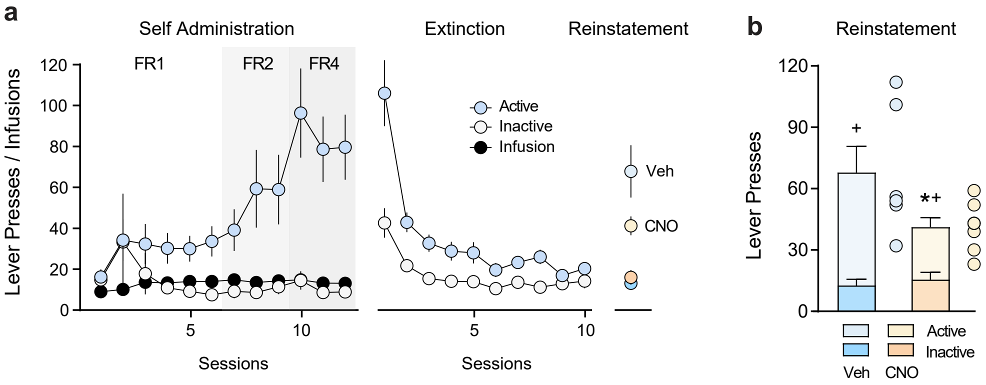

Supplementary Figure 5. Stimulating neuronal activity in the IL→NAshell pathway reduces cued heroin relapse after extinction training. (a) Self administration, extinction, and cued reinstatement of heroin seeking in a standard model. This study was designed to mimic our previously published work on cocaine [9]. Hence, all methodology was identical to that study except that heroin was substituted for cocaine (at the same dose and infusion parameters reported in the main text), and an increase in FR requirement was imposed during self-administration as shown. Cues were thus presented on an FR2 schedule during the relapse test. (b) Stimulation of the IL→NAshell pathway using the Gq-DREADD reduces cued heroin relapse after extinction (two-way ANOVA lever x treatment interaction $F_{(1,11)} = 6.54$, $p = 0.03$, Bonferroni post-hoc for treatment: $p = 0.02$, vehicle lever: $p = 9.03 \times 10^{-5}$, and CNO lever: $p = 0.02$). CNO: DREADD ligand clozapine-N-oxide. Vehicle: n = 6, CNO: n = 7 rats. + $p < 0.05$ comparing between active and inactive levers, * $p < 0.05$ comparing between vehicle and CNO. Data are represented as mean ± S.E.M.
