# Supplementary material for: A common limiter circuit for opioid choice and relapse identified in a rodent addiction model
Source: Nat Commun. 2021 Aug 9;12:4788. doi: 10.1038/s41467-021-25080-x (PMC8352904; doi:10.1038/s41467-021-25080-x)
Supplement: Supplementary file 2 — Reporting Summary [file 41467_2021_25080_MOESM2_ESM.pdf]

nature research

Corresponding author(s): Jamie Peters

Last updated by author(s): 06/25/2021

Reporting Summary

Nature Research wishes to improve the reproducibility of the work that we publish. This form provides structure for consistency and transparency in reporting. For further information on Nature Research policies, see our Editorial Policies and the Editorial Policy Checklist.

Statistics

For all statistical analyses, confirm that the following items are present in the figure legend, table legend, main text, or Methods section.

n/a	Confirmed	
☐	☒	The exact sample size (*n*) for each experimental group/condition, given as a discrete number and unit of measurement
☐	☒	A statement on whether measurements were taken from distinct samples or whether the same sample was measured repeatedly
☐	☒	The statistical test(s) used AND whether they are one- or two-sided<br>*Only common tests should be described solely by name; describe more complex techniques in the Methods section.*
☐	☒	A description of all covariates tested
☐	☒	A description of any assumptions or corrections, such as tests of normality and adjustment for multiple comparisons
☐	☒	A full description of the statistical parameters including central tendency (e.g. means) or other basic estimates (e.g. regression coefficient) AND variation (e.g. standard deviation) or associated estimates of uncertainty (e.g. confidence intervals)
☐	☒	For null hypothesis testing, the test statistic (e.g. *F*, *t*, *r*) with confidence intervals, effect sizes, degrees of freedom and *P* value noted<br>*Give P values as exact values whenever suitable.*
☒	☐	For Bayesian analysis, information on the choice of priors and Markov chain Monte Carlo settings
☒	☐	For hierarchical and complex designs, identification of the appropriate level for tests and full reporting of outcomes
☒	☐	Estimates of effect sizes (e.g. Cohen's *d*, Pearson's *r*), indicating how they were calculated

Our web collection on statistics for biologists contains articles on many of the points above.

Software and code

Policy information about availability of computer code

Data collection	Med PC IV (Med Associates) was used for the acquisition of behavioral data, Olympus FV1200 software (FV10-ASW; version 04.10) was used for the acquisition of confocal images
Data analysis	MATLAB (Mathworks, version R2019b) was used the analysis of behavioral economics data, curve fitting, and principal component analysis. Excel (Microsoft Office 365) was used for the analysis of behavioral data and structural analyses. Huygens (version 17.10; SVI) was used to deconvolve image stacks prior to image analysis. Imaris (version 9.0.1; Bitplane) was used for structural analyses and cell counts.

For manuscripts utilizing custom algorithms or software that are central to the research but not yet described in published literature, software must be made available to editors and reviewers. We strongly encourage code deposition in a community repository (e.g. GitHub). See the Nature Research guidelines for submitting code & software for further information.

Data

Policy information about availability of data

All manuscripts must include a data availability statement. This statement should provide the following information, where applicable:

- Accession codes, unique identifiers, or web links for publicly available datasets
- A list of figures that have associated raw data
- A description of any restrictions on data availability

The datasets generated during and/or analysed during the current study are available from the corresponding author on reasonable request.

April 2020

Field-specific reporting

Please select the one below that is the best fit for your research. If you are not sure, read the appropriate sections before making your selection.

☒ Life sciences ☐ Behavioural & social sciences ☐ Ecological, evolutionary & environmental sciences

For a reference copy of the document with all sections, see nature.com/documents/nr-reporting-summary-flat.pdf

Life sciences study design

All studies must disclose on these points even when the disclosure is negative.

Sample size	Sample size was determined based on previous studies utilizing the same methodology and/or targeting the same brain regions. These include Augur et al. (2016, Journal of Neuroscience; PMID: 27683912), Gianotti et al. (2018, Journal of Neuroscience; PMID: 30185459) and Pardo-Garcia et al. (2019, Journal of Neuroscience; PMID: 30622165).
Data exclusions	Rats were excluded from the study based on (1) misplaced intracranial cannulas or virus injections or the absence of virus expression, (2) unstable behavior, (3) catheter failure or the development of sickness during the study. Exclusion criteria were established a priori, before experiments were conducted.
Replication	Attempts at replicating the data were successful as described here. The main findings of the study (subpopulations of rats prefer heroin over food reward and a selective neural circuit is capable of limiting drug choice; Figure 2a) were replicated in a second independent cohort using an alternative method (pharmacological inactivation of the region that contains the cell bodies for this circuit; Figure 3b). Another main finding (the circuit also limits drug seeking during relapse; Figure 3d,4e) was not replicated between these different methods likely due to the involvement of competing neural circuits (cell bodies) located in the targeted brain region using the pharmacological inactivation strategy. Of note, while these were not exact replications per se due to the use of different methodologies, the consistency of the finding on heroin choice increases the rigor of this study. No attempt was made to replicate other findings reported in this study (behavioral economics analyses, spine analyses; Figures 1, 6).
Randomization	Rats were randomly assigned to counterbalanced treatment groups for testing based on prior behavioral responding. In addition, a crossover design was used for behavioral experiments such that all rats were tested twice for each test (once with an active manipulation, and once with a control treatment)
Blinding	Personnel running the behavioral experiments and histological validation of intracranial placements were blind to experimental manipulations and experimental outcomes. Images for cell counts and structural analyses were acquired blind and data was analyzed in a blinded manner.

Reporting for specific materials, systems and methods

We require information from authors about some types of materials, experimental systems and methods used in many studies. Here, indicate whether each material, system or method listed is relevant to your study. If you are not sure if a list item applies to your research, read the appropriate section before selecting a response.

Materials & experimental systems

n/a	Involved in the study	
☐	☒	Antibodies
☒	☐	Eukaryotic cell lines
☒	☐	Palaeontology and archaeology
☐	☒	Animals and other organisms
☒	☐	Human research participants
☒	☐	Clinical data
☒	☐	Dual use research of concern

Methods

n/a	Involved in the study	
☒	☐	ChIP-seq
☒	☐	Flow cytometry
☒	☐	MRI-based neuroimaging

Antibodies

Antibodies used	Primary antibodies used included rabbit anti-cFos (1:2000, Millipore Cat# ABE457, RRID: AB_2631318), chicken anti-mCherry (1:5000, LifeSpan, Cat# LS-C204825, RRID: AB_2716246). Secondary Alexa-fluor conjugated antibodies (1:500; Jackson ImmunoResearch Labs) used were donkey anti-Chicken 488 (Cat# 703-585-155, RRID:AB_2340377), donkey anti-chicken 594 (Cat# 703-585-155, RRID:AB_2340377), donkey anti-Rabbit 680 (Cat# 711-625-152, RRID:AB_2340627) and donkey anti-Rabbit 488 (Cat# 711-545-152, RRID:AB_2313584).
Validation	cFos antibody validated and titrated in our lab using tissue with known expression pattern of Fos protein and initial concentration chosen based on literature (RRID: AB_2631318). mCherry antibody concentration chosen based on literature (RRID: AB_2716246) and validated in our lab using positive and negative control tissue.

Animals and other organisms

Policy information about studies involving animals; ARRIVE guidelines recommended for reporting animal research

Laboratory animals	Age-matched (P55-60) male and female Wistar rats were used. Rats were purchased from Charles River (Raleigh, NC)
Wild animals	No wild animals were used in the study
Field-collected samples	No field-collected samples were used in the study
Ethics oversight	All experiments were approved by the Institutional Animal Care and Use Committee at the University of Colorado Denver, Anschutz Medical Campus. Rats were housed, and experiments were conducted in a AAALAC accredited facility.

Note that full information on the approval of the study protocol must also be provided in the manuscript.
